# The Interaction between hERG1 and β1 Integrins Modulates hERG1 Current in Different Pathological Cell Models

**DOI:** 10.3390/membranes12111162

**Published:** 2022-11-18

**Authors:** Ginevra Chioccioli Altadonna, Alberto Montalbano, Jessica Iorio, Andrea Becchetti, Annarosa Arcangeli, Claudia Duranti

**Affiliations:** 1 Department f Medical Biotechnologies, University of Siena, Strada delle Scotte, 53100 Siena, Italy; 2 Department of Experimental and Clinical Medicine, University of Firenze, Viale G.B. Morgagni 50, 50134 Firenze, Italy; 3 Department of Biotechnology and Biosciences, University of Milano-Bicocca, Piazza della Scienza 2, 20126 Milano, Italy

**Keywords:** hERG1, adhesion molecules, ion channels, fibronectin, macromolecular complexes, ECM proteins, neuroblastoma

## Abstract

Ion channels are implicated in various diseases, including cancer, in which they modulate different aspects of cancer progression. In particular, potassium channels are often aberrantly expressed in cancers, a major example being provided by hERG1. The latter is generally complexed with β1 integrin in tumour cells, and such a molecular complex represents a new druggable hub. The present study focuses on the characterization of the functional consequences of the interaction between hERG1 and β1 integrins on different substrates over time. To this purpose, we studied the interplay alteration on the plasma membrane through patch clamp techniques in a cellular model consisting of human embryonic kidney (HEK) cells stably transfected with hERG1 and in a cancer cell model consisting of SH-SY5Y neuroblastoma cells, endogenously expressing the channel. Cells were seeded on different substrates known to stimulate β1 integrins, such as fibronectin (FN) for HEK-hERG1 and laminin (LMN) for SH-SY5Y. In HEK cells stably overexpressing hERG1, we observed a hERG1 current density increase accompanied by V_rest_ hyperpolarization after cell seeding onto FN. Notably, a similar behaviour was shown by SH-SY5Y neuroblastoma cells plated onto LMN. Interestingly, we did not observe this phenomenon when plating the cells on substrates such as Bovine Serum Albumin (BSA) or Polylysine (PL), thus suggesting a crucial involvement of ECM proteins as well as of β1 integrin activation.

## 1. Introduction

The altered function of ion channels contributes to various diseases including cancer [1,2]. Thanks to their key role in sensing and integrating signals from the extracellular context, these proteins are emerging as particularly relevant elements in cancer, mediating interactions between tumour cells and their microenvironment. Such interaction regulates neoplastic progression events, such as cell proliferation, survival, invasiveness and pro-angiogenetic programs [3,4]. Moreover, being primarily localized in the plasma membrane, ion channels represent one of the few druggable molecular classes and they are increasingly being recognized as novel and valuable molecular targets for antineoplastic therapy [5]. The mechanisms through which ion channels contribute to tumour progression are numerous. K^+^ channels, for instance, allow uncontrolled tumour cell proliferation by setting the membrane potential (V_rest_) to depolarized values [6].

Potassium channels are functionally expressed in several somatic cancer cell lines and primary tumours [7,8,9] and their expression has been detected in many tumour cell lines, such as Pancreatic Ductal Adenocarcinoma (PDAC) tumour cells and neuroblastoma cell lines [10,11]. Overall, ion channels might be considered as novel cancer biomarkers and, potentially, as good targets for antineoplastic therapy. Particularly interesting is hERG1, which is a voltage-gated potassium channel, also known as Kv11.1, physiologically expressed in cardiac myocytes, neurons, smooth muscles of different organs, and neuroendocrine cells [12]. It underlies the rapid delayed rectifier current in the heart that is essential for repolarization of the cardiac action potential and, consequently, normal cardiac electrical activity and rhythm [13]. In contrast to other Kv channels, hERG1 displays unusual gating characteristics, which include slow activation and rapid voltage-dependent inactivation. This channel is aberrantly expressed in many primary human cancers, such as glioma, neural crest-derived tumours (neuroblastoma and melanoma) and a variety of carcinomas (i.e., PDAC) and leukaemia, where it regulates many stages of tumorigenesis: from cell proliferation and survival to cell invasiveness and neo-angiogenesis [3,4,5,6,7,8,12]. Notably, such pleiotropic effects are not necessarily the same, even in closely related cancers [12]. Moreover, Kv11.1 regulates several aspects of cell physiology by its interaction with integrin receptors. This regulation depends on both the formation of a macromolecular complex with the β1-integrin subunit and on signalling crosstalk between the channel and integrins [14]. These mechanisms, indeed, control downstream signalling pathways, such as tyrosine kinases and GTPases [5].

It has already been shown [15] that half of the PDAC cell lines express this channel at very high levels and that hERG1 is present in the primary tumour. This channel starts to be expressed in the Pancreatic Intraepithelial Neoplasia (PaIN) and its expression is increased with tumour progression [16]. Moreover, it has been demonstrated that in the PDAC, hERG1 is coexpressed and interacts with different membrane receptors, such as EGFR and β1-integrins [17]. The molecular interaction between hERG1 and β1-integrins influences the channel activity. It is a gating-dependent complex formation hindered by the open state of hERG1 [3,4].

Regarding neuroblastoma cells, our group previously reported that a long-lasting hERG1 activation occurs after integrin-mediated adhesion, which is associated with the induction of neurite extensions and differentiation [18,19]. No other K^+^ currents endogenously expressed were increased after cell adhesion in these cells [20]. Subsequently, we demonstrated that the β1 integrin subunit coprecipitates with hERG1 in SH-SY5Y neuroblastoma cells [21].

**Figure 1:**
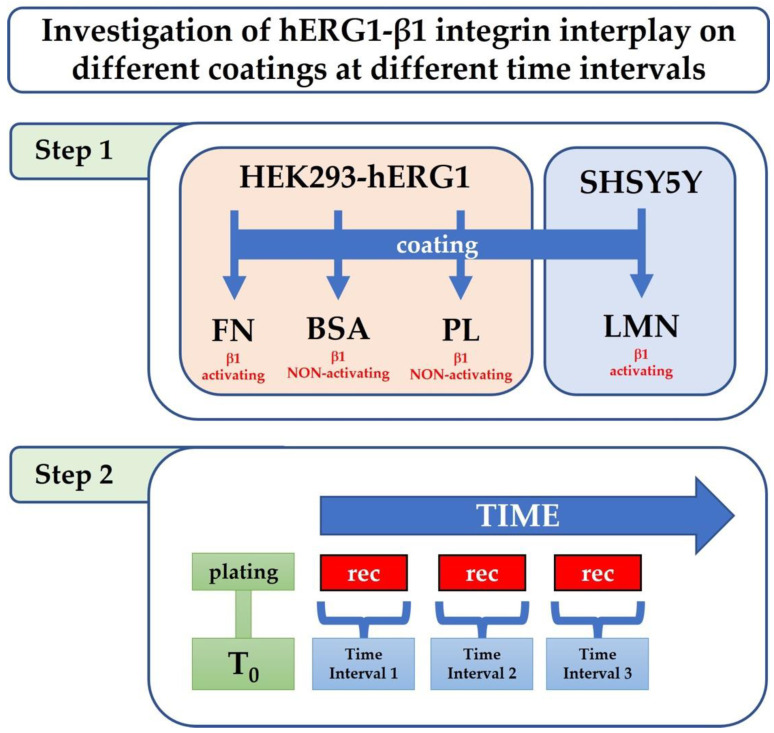
**hERG1-β1 integrin investigation workflow.** Workflow of the experimental design. Cell lines were plated on different coatings, activating and non-activating the β1 integrin. Electrophysiological recordings were performed at different time intervals.

To further examine these interactions over time, we studied and characterized through patch clamp experiments the hERG1-β1 integrin interplay on different coatings (FN, LMN and BSA, PL substrates, which do not activate β1 integrins) at different intervals of time from the cells. In particular, we tested a cellular model consisting of the human embryonic kidney (HEK) cells stably transfected with hERG1 on FN, BSA and PL. We then validated the role of this interplay in the tumour cell line SH-SY5Y on LMN as a pathophysiological model (Figure 1). We used FN for HEK-hERG1 cells and LMN for neuroblastoma SH-SY5Y cells for their different integrins’ expression profiles [19,20].

## 2. Materials and Methods

### 2.1. Cell Culture

HEK293 cells were obtained from the American Type Culture Collection (ATCC). SH-SY5Y cells were a kind gift from Prof. P. Defilippi (Department of Molecular Biotechnology and Health Sciences, University of Turin, Turin, Italy). Cells were routinely cultured at 37 °C with 5% CO_2_ in a humidified atmosphere, in Dulbecco’s modified Eagle’s Medium (DMEM; Euroclone) supplemented with 4% L-Glut and 10% fetal bovine serum (FBS, Fetal Bovine Serum EU-Approved, Euroclone, Pero, Italy). We certify that all the cells used in the present study were routinely screened for Mycoplasma contamination, and only Mycoplasma negative cells were used. HEK293 cells expressing the hERG1 construct (HEK hERG1) were prepared as previously described [3] and maintained in complete culture medium supplemented with either 0.8 mg/mL (for HEK293 cells) of Geneticin (G418, Thermo Fisher Scientific, Waltham, MA, USA). Time zero was defined as the time point corresponding to the cell seeding.

### 2.2. Coatings

Fibronectin (Sigma-Aldrich, Darmstadt, Germany, human plasma) coating was performed following the standard protocol provided with the product. In particular, FN was diluted in sterile PBS (Euroclone) at 5 µg/cm^2^ concentration. The culture surface was coated with a minimal volume (1 mL for 35 mm Petri dishes). The dishes were left air-drying for 1 h at room temperature before introducing cells and medium.

Laminin (Sigma-Aldrich, Engelbreth-Holm-Swarm murine sarcoma basement membrane) coating was performed in accordance with the information provided with the product. More in detail, LMN was diluted in sterile PBS at 0.02% concentration to coat the culture surface (1 mL for 35 mm Petri dishes) and left air-drying for 1 h before plating cells.

Heat-inactivated BSA was prepared by heating a BSA (Sigma-Aldrich) solution (25 mg/mL) in sterile PBS (Euroclone) at 70 °C for 1 h. Coating of culture dishes was performed by adding the BSA at 0.25 mg/mL in DMEM at 37 °C for 1 h (1 mL for 35 mm Petri dishes) before seeding cells.

Polylysine (Sigma-Aldrich, Poly—L-Lysine solution 0.01%) coating was performed following the standard protocol for the product. The culture surface was coated with 1 mL/25 cm^2^ and rocked gently to ensure an even coating. After 5 min the solution was removed by aspiration and the surface was washed with sterile water. The dishes were left to air-dry at least 2 h before introducing cells and medium.

### 2.3. Patch Clamp Recording

On the experimental day, HEK293 cells were detached, resuspended in DMEM + HI BSA and seeded on FN-coated, BSA-coated, PL-coated or LMN-coated Petri dishes for patch clamp experiments. Electrophysiological recordings were performed at room temperature (~25 °C) in the whole-cell configuration of the patch clamp technique, at different time intervals (minutes) after cell seeding (i.e., T_5–15_, T_30–45_, T_60–90_), during which cells were maintained in an incubator at 37 °C, 5% CO_2_. The beforementioned time points are the intervals of time (expressed in minutes) ranging from the cell seeding to the time in which the recordings were acquired. The patch pipettes were pulled from borosilicate glass capillary tubes to a resistance of 4–5 MΩ. Capacitances were manually compensated after the reaching of a stable gigaseal. The cell capacitances of cells were 34.8 ± 0.6 pF. Experimental protocols and data acquisition were performed with the Multiclamp 700 A or Multiclamp 1D amplifiers and pCLAMP 9.2 software (Molecular Devices, Sunnyvale, CA, USA) was used for data analysis. The hERG1 inward tail currents were recorded with a 25 KHz sampling rate and a 0.2 kHz low-pass filter. Cells’ identification and patch were performed at 40× magnification with a Nikon Eclipse TE300 microscope (Nikon Instruments Inc., Amstelveen, The Netherlands), equipped with a Photometrics CoolSNAP CF camera (Teledyne Photometrics, Tucson, AZ, USA). Cell membrane potentials were held at −80 mV, and hERG1 inward tail currents were elicited using preconditioning holding potential ranging from 0 mV to −100 mV (10 mV step increment) followed by 1 s hyperpolarizing step (−120 mV) with an intersweep interval of 15 s. The internal pipette solution contained (in mM): 130 K^+^ aspartate, 10 NaCl, 4 CaCl_2_, 2 MgCl_2_, 10 Hepes–NaOH, 10 EGTA, pH 7.3. The external solution contained (in mM): 130 NaCl, 5 KCl, 2 CaCl_2_, 2 MgCl_2_, 10 HEPES, 5 glucose (E_K_ = −80 mV), pH of 7.4. Resting membrane potential (V_rest_) values were measured in I-0 mode.

### 2.4. Statistical Analysis

Parametric tests were used for statistical analysis, i.e., unpaired *t*-test. In particular, for comparison of data groups, one-way ANOVA test with Dunnett’s multiple comparison post hoc test was used if distribution was normal (*p* > 0.05) when tested with the D’Agostino and Pearson omnibus normality test (i.e., comparison of CD_hERG1_, V_rest_ and V_1/2_ values in SH-SY5Y). Two-way ANOVA test with Tukey’s multiple comparison post hoc test was used for comparisons among different coatings over time (i.e., CD_hERG1_, V_rest_ and V_1/2_ on FN, BSA, PL). Data are reported as mean ± SEM. Statistical analysis was performed using Prism 9 software (GraphPad Software, San Diego, CA, USA).

## 3. Results

### 3.1. β1 Integrin Activation Leads to hERG1 Current Density Increase and Resting Membrane Potential Hyperpolarization: A Kinetic Alteration?

To investigate the hERG1/β1 integrin interplay over time, HEK-hERG1 cells were seeded onto FN, BSA and PL, and electrophysiological recordings were performed at different time intervals after cell seeding to monitor the maximal hERG1 current density (CD_hERG1_) and the resting potential (V_rest_) values. We observed different behaviours related to the coating over time. First, we monitored the CD_hERG1_ at different intervals of time, the latter expressed in minutes, ranging from the cell seeding to the time in which the recordings were acquired.

**Figure 2:**
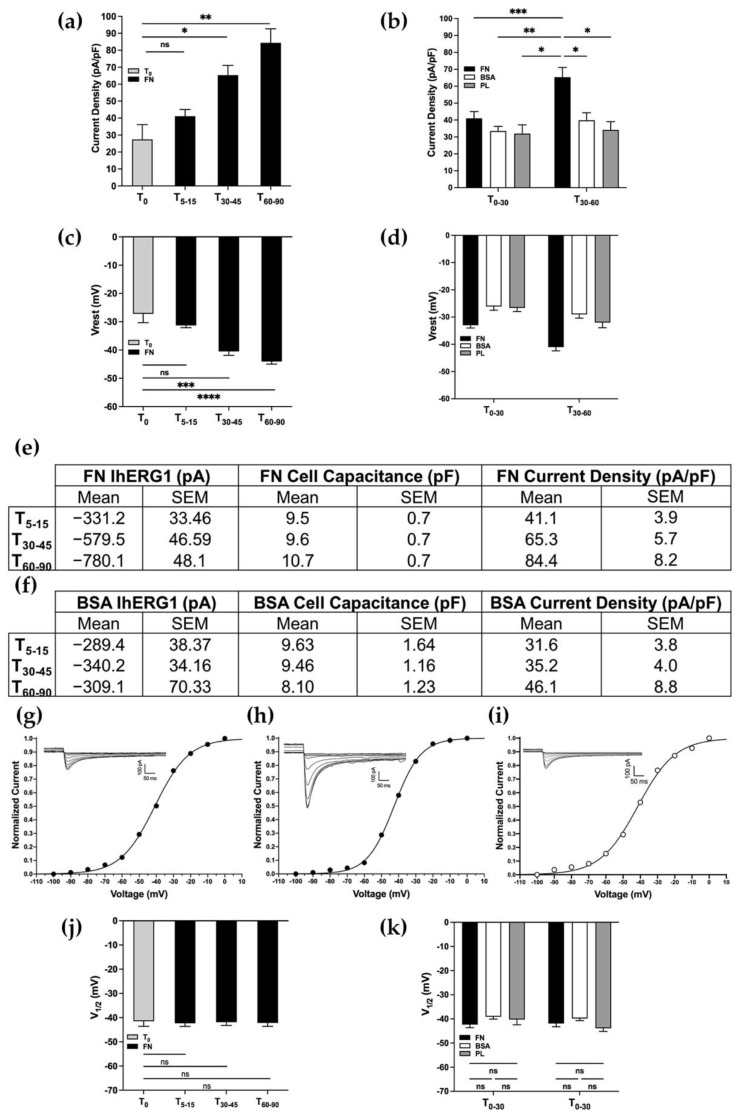
**β1 integrin activation by FN leads to hERG1 current density increase and resting membrane potential hyperpolarization**. (**a**,**c**,**j**) Bar graph to compare CD_hERG1_, V_rest_ and activation V_1/2_ recorded in cells seeded on FN at different time intervals after cell seeding and compared to the T_0_ (total number of analysed cells: (**a**,**c**) = 133; (**j**) = 66). (**b**) Bar graph to compare CD_hERG1_ recorded in cells seeded on FN (black), BSA (white) and onto PL (grey; total number of analysed cells = 126). (**d**) Bar graph to compare V_rest_ recorded in cells seeded on FN (black), BSA (white) and onto PL (grey; total number of analysed cells = 126). (**e**) Table reporting mean values of I_hERG1_, cell capacitance and current density of HEK-hERG1 cells plated onto FN and BSA (**f**) over time. (**g**–**i**) Patch clamp inward current traces and activation curves of a representative cell at T0, after 90 min of incubation onto FN and after 90 min of incubation onto BSA. (**k**) Bar graph to compare activation V_1/2_ recorded in cells seeded on FN (black), BSA (white) and onto PL (grey; total number of analysed cells = 89). All data shown are mean values ± s.e.m. obtained from at least four cell patch clamp experiments. All the aforementioned time points are the intervals of time (expressed in minutes) ranging from the cell seeding to the time in which the recordings were acquired. For statistical significance, two-way ANOVA test with Tukey’s multiple comparison post hoc test was used. * *p* < 0.0332; ** *p* < 0.0021, *** *p* < 0.0002 and **** *p* < 0.0001.

As shown in Figure 2a, we observed an increase in the current density reaching a peak at T_60–90_, doubling the initial value (T_0_ = 27.4 ± 8.7 pA/pF; T_60–90_ = 84.4 ± 8.2 pA/pF). We then compared the FN coating with the BSA and the PL, in two different intervals of time, T_0–30_ and T_30–60_, and it clearly emerged that only the adhesion onto FN determined a significant increase in the CD_hERG1_ at T_60–90_ as shown in Figure 2b. The adhesion onto FN, indeed, elicited a CD_hERG1_ increase that was not observed in cells seeded onto BSA or PL (Figure 2b; p_T0–30FNvsBSA_ = 0.918; p_T0–30FNvsPL_ = 0.938; p_T0–30BSAvsPL_ > 0.999; p_T30–60FNvsBSA_ = 0.028; p_T30–60FNvsPL_ = 0.030; p_T30–60BSAvsPL_ = 0.996). In parallel with the CD_hERG1_ increase, the average V_rest_ of FN-seeded HEK-hERG1 cells was hyperpolarized from the initial values (T_0_ = −24.1 ± 1.4 mV; T_60–90_ = 44.1 ± 0.9 mV) and it was significantly different from what we observed in the cells seeded onto BSA and PL for all the time intervals monitored, as shown in Figure 2c,d (Figure 2c: p_T0–T5-15_ = 0.113; p_T0–T30–45_ = 0.0002; p_T0–T60–90_ < 0.0001; 1e: p_T0–30_ = 0.0005; p_T30–60_ < 0.0001; Figure 2d: p_T0–30FNvsBSA_ = 0.008; p_T0–30FNvsPL_ = 0.130; p_T0–30BSAvsPL_ > 0.999; p_T30–60FNvsBSA_ < 0.001; p_T30–60FNvsPL_ = 0.011; p_T30–60BSAvsPL_ = 0.912). Moreover, the different coatings did not affect the cell capacitance over time (Figure 2e,f; two-way ANOVA, *p* = 0.476).

To better characterize this phenomenon, we investigated whether or not the current density increase and the V_rest_ hyperpolarization were due to biophysical or kinetic modifications of the channel. In particular, we analysed whether the steady-state activation curves of hERG1 were modified by cell adhesion onto FN when compared to the one seeded onto BSA and PL. In both cases, we did not observe any significant modification of V_1/2_ related to the coating (Figure 2j,k; Figure 2j: p_T0/T5–15_ = 0.966; p_T0/T30–45_ = 0.995; p_T0/T60–90_ = 0.971; Figure 2k: p_T0–30FNvsBSA_ = 0.480; p_T0–30FNvsPL_ = 0.933; p_T0–30BSAvsPL_ = 0.988; p_T30–60FNvsBSA_ = 0.791; p_T30–60FNvsPL_ = 0.936; p_T30–60BSAvsPL_ = 0.264). However, in agreement with previous work [3], the estimated V_1/2_ of activation was around −40/−50 mV. Representative patch clamp traces and I/V curves obtained in HEK-hERG1 cells seeded onto either FN or BSA at different time intervals are reported in Figure 2g–i and clearly show that the I_hERG1_ increase only occurs in the first condition and that it is not accompanied by a V_1/2_ modification.

### 3.2. hERG1-β1 Interaction in a Neuroblastoma Cell Line

**Figure 3:**
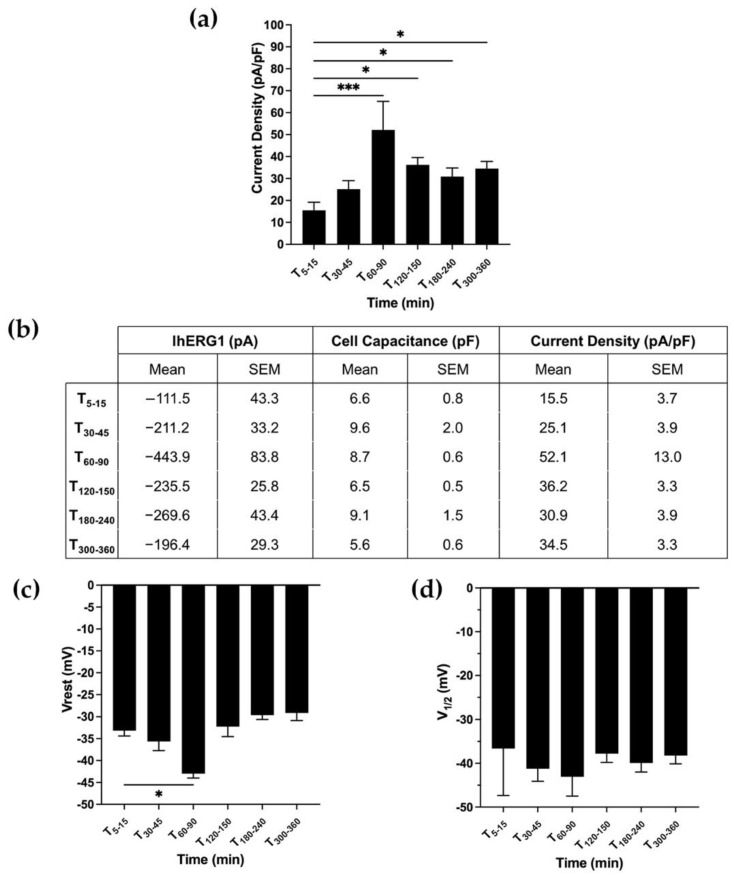
**The pathophysiological model of SH-SY5Y.** (**a**) Bar graph of CD_hERG1_ in SH-SY5Y WT cells seeded onto LMN and recorded at different time intervals after cell seeding (total number of analysed cells = 32). (**b**) Table showing values of IhERG1, cell capacitance and current density over time. Data are mean values ± s.e.m. obtained from at least three cell patch clamp experiments. (**c**) Bar graph of the V_rest_ in SH-SY5Y WT cells seeded onto LMN (total number of analysed cells = 32). (**d**) Bar graph of activation V_1/2_ of SH-SY5Y cells plated onto LMN at different time intervals after cell seeding (total number of analysed cells = 32). All data shown are mean values ± s.e.m. obtained from at least four cell patch clamp experiments. All the aforementioned time points are the intervals of time (expressed in minutes) ranging from the cell seeding to the time in which the recordings were acquired. For statistical significance, ordinary one-way ANOVA with Dunnett’s multiple comparisons test was applied after applying the D’Agostino and Pearson omnibus normality test. * *p* < 0.0332; ** *p* < 0.0021, *** *p* < 0.0002 and **** *p* < 0.0001.

Next, we further investigated CD_hERG1_, V_rest_ and V_1/2_ dynamics over time in SH-SY5Y neuroblastoma cells, in which we previously demonstrated that integrin stimulation determines a long-lasting activation of I_hERG1_ and that this is the only endogenously expressed potassium current [18]. Neuroblastoma cells, therefore, represent a pathophysiological model where the hERG1/b1 interplay is investigated to assess similarities or differences with the HEK-hERG1 transfected model. SH-SY5Y, indeed, was the first model in which the hERG1 expression and the effect of laminin were detected by our group [19] but its dynamic over time needed to be better investigated. Longer time intervals were monitored due to SH-SY5Y due to their longer spreading time compared to HEK-hERG1 [20,22,23]. Electrophysiological recordings in cells plated on the ECM protein laminin (LMN) at different time points (T_5–15_, T_30–45_, T_60–90_, T_120–150_, T_180–240_ and T_300–360_ min after plating) showed a CD_hERG1_ 3.4-fold increase (at T_60–90_: 52.1 ± 13.0 pA/pF) from the initial value (15.5 ± 3.7 pA/pF). Afterwards, CD_hERG1_ decreased, attaining values around 30–35 pA/pF (Figure 3a; ordinary one-way ANOVA with Dunnett’s multiple comparisons test, *p* = 0.002). As we observed in HEK cells stably overexpressing hERG1, the current density increase was accompanied by a V_rest_ hyperpolarization, reaching the peak after 90 min from cell seeding (∆ = 10 mV) and then diminishing to the initial values around −30 mV (Figure 3c). Because of the similarities with what was observed in HEK-hERG1 cells, we also monitored the activation V_1/2_ values of neuroblastoma cells plated onto laminin. Once again, no significant differences occurred (Figure 3c; ordinary one-way ANOVA with Dunnett’s multiple comparisons test, *p* = 0.511).

## 4. Discussion

The regulatory interaction between cell–cell or cell–substrate adhesion receptors and ion transport was identified several decades ago. It is now known that integrins mediate adhesion but are also deeply involved in ion fluxes’ modulation [24]. The dynamic of macromolecular complexes in response to tumorigenesis is still unclear, though. Although ample evidence indicates that the engagement of integrins can promote potassium efflux by both excitable and non-excitable cells, Brown et al. (2008) speculate that the activation state of integrins is dynamically regulated by changes in the transmembrane potential [25]. The hypothesis is that the co-association between integrins and voltage-gated potassium channels, such as hERG1, would lead to a conformational change in the voltage sensor that is then relayed or transmitted to the integrins themselves [26,27]. This could trigger the integrins’ downstream signalling (i.e., FAK activation). Our group previously reported that it is possible that hERG1 activation occurring early during cell adhesion has a role in stimulating FAK phosphorylation and the ensuing signalling pathways, whereas the late formation of the macromolecular complex progressively shifts the hERG1 channel population toward the non-conducting state [3]. Those findings suggest that ion flux and hERG1 voltage-related conformation are crucial for FAK phosphorylation and in turn for complex-related signalling, being consistent with Brown’s hypothesis.

Although understanding of macromolecular complexes and their clinical relevance has increased significantly in recent years, numerous topics for further research remain. Importantly, our results showed the functional response of hERG1 to different coatings, such as FN, PL, BSA and LMN in both a reconstituted model (HEK-hERG1) and in a physiological one (SH-SY5Y). Our electrophysiological recordings showed that *β*1 integrin activation, triggered by cell adhesion onto ECM proteins (FN and LMN), induces a double effect on hERG1 channels. In particular, we observed an increase in the hERG1 current amplitude together with the V_rest_ hyperpolarization. Interestingly, no such phenomenon was observed when cells were plated onto BSA and PL. However, our hypothesis of a biophysical alteration induced by the hERG1-*β*1 complex formation was proved wrong. In fact, no significant differences were observed regarding the activation V_1/2_, thus excluding a biophysical modification of hERG1. Therefore, our work is in accordance with what is described above, but it also highlights the crucial role of ECM proteins in the *β*1 integrin activation and in the macromolecular complex formation.

In addition to this, it is well known that the depolarized V_rest_, found in cancer cells, could be regarded as a “sustaining proliferative signal” that instructs cells to rapidly advance in the cell cycle [28]. However, numerous studies highlighted that membrane hyperpolarization at the G1/S checkpoint is generally required for S phase initiation. For instance, depolarizing the cell membrane halts the G1/S progression of various cell lines, such as in glia [29], mouse neuroblastoma cells [30], MCF-7 human breast cancer cells [31] and lymphocytes [32,33,34]. Some cell lines require a relatively hyperpolarized membrane potential during the S phase [30,31], whilst others a more depolarized V_rest_, such as human neuroblastoma cells [7]. Fluctuations of K^+^ concentration influence the membrane potential setting during the cell cycle. A transient decrease in K^+^ efflux before entering the G2 phase and a relatively high level of K+ efflux during the M phase, indeed, has been found in both mouse neuroblastoma and Ehrlich ascites cells [30,35].

Voltage-gated potassium channels, then, are involved in the regulation of proliferation and membrane potential. It is well known that hERG channels are expressed at early developmental stages in the neural crest, central nervous system, dorsal root ganglion (DRG) and skeletal muscle, and are replaced by a classic inward rectifier K^+^ current (IKir) later in development [36,37]. Moreover, as already highlighted, hERG channels are upregulated in a number of cancers [8] and their current increases tumour cell proliferation [3,4,5,6,7,8,12,38,39]. The activity of I_hERG1_ itself is cell cycle-dependent [7], suggesting a complex but crucial relationship between I_hERG1_, V_rest_ and proliferation. The mechanisms underlying ion channel-dependent proliferation of cancer cells include possible non-conducting, direct interactions between ion channels and other pro-proliferative signalling mechanisms [28]. For instance, coexpression of hERG and tumour necrosis factor receptor 1 (TNFR1) has been found at the cell membrane of both SKBR3 and SH-SY5Y cell lines, suggesting a hERG role in recruiting TNFR1 to the membrane, therefore enhancing TNF-α-induced cancer cell proliferation [39].

It is evident that in such a scenario, unveiling of the dynamics of the interaction of the macromolecular complexes between ion channels and receptors and their effect on ion currents and molecular signalling is urgently needed. Such findings, indeed, would give us important opportunities for new pharmacological targeting. The latter will need versatile tools, such as bispecific antibodies, as they offer the unique advantage of simultaneously binding two or more proteins, impacting the downstream signalling [6,40,41,42], which could be achieved by targeting different conformational states.

## 5. Conclusions

In conclusion, our study aimed to functionally characterize the hERG1/β1 interplay over time. Our findings showed a CD_hERG1_ increase together with the V_rest_ hyperpolarization in response to cell adhesion onto ECM proteins, such as fibronectin for HEK-hERG1 cells and laminin for SH-SY5Y cells. Our first hypothesis of a biophysical modification of the channel was proved wrong, opening the way for further investigations regarding the pathways involved and the mechanisms hiding behind crucial phenomenon, which could open the way to new pharmacological strategies for targeting.

## Data Availability

Not applicable.
